# A thematic content analysis of the structure and effects of good doctor abilities in China

**DOI:** 10.1186/s12913-024-11145-2

**Published:** 2024-07-16

**Authors:** Zhongguang Yu, Xiang Hu, Hongjin Li, Ning Hu, Yanping Li

**Affiliations:** 1 College of Economics and Management, Wuhan University, Wuhan, Hubei 430072 China; 2 Respiratory Center, China-Japan Friendship Hospital, Beijing, 100029 China; 3 Business School, Hubei University, Wuhan, Hubei 430062 China; 4 Tongji Medical College, Huazhong Science and Technology University, Wuhan, Hubei 430030 China; 5 School of Management, Beijing University of Chinese Medicine, Beijing, 102488 China

**Keywords:** The good doctor, Abilities structure, Effects, Thematic content analysis

## Abstract

**Background:**

The efforts to explore and build the structure of good doctor abilities are important because they help improve the quality of education for medical students and better standardize the working performance of doctors. However, at present, no worldwide standards for such a structure have been established. In this study, we endeavoured to map the structure of good doctor abilities and identify their effects.

**Methods:**

With a focus on China, a thematic content analysis was adopted in this study to analyse the personal profiles of 50 widely recognized good doctors. NVivo11 software was used.

**Results:**

The Structure and Effects of Good Doctor Abilities in China model was proposed, and interpretations were made based on AMO theory. Good doctor abilities fall within six categories: rigorous clinical thinking, skilled in diagnosis and therapy, clinical empathy, continuous learning and innovation, enhancing and sharing experiences, and communication and coordination. These abilities have positive impacts on doctors’ work performances and social benefits by encouraging good behaviours, ultimately promoting the sustainable development of the hospitals where they serve.

**Conclusions:**

In this study, we established a model of the structure and effects of good-doctor abilities in China and interpreted its mechanism, innovation and theory diversification in “good-doctor” research. Moreover, this study has practical significance because it provides systematic and well-targeted criteria for improving the professionalism of doctors, promoting more good doctor behaviours, providing guidance for regulating doctors’ conduct and providing a reference for medical education and working performance reviews worldwide.

**Supplementary Information:**

The online version contains supplementary material available at 10.1186/s12913-024-11145-2.

## Background

“Doctor” is a well-respected profession. Doctors fight against human afflictions, heal the wounded, and rescue the dying, making great contributions to the well-being of Mankind. According to the *“Global Strategy on Human Resources for Health: Workforce 2030”* by the WHO, there will be more than 13.8 million doctors worldwide looking after our health and preventing diseases by the year 2030 [1]. One study conducted during the COVID-19 pandemic indicated that “health care workers spend 16 hours each day on average caring for patients infected by COVID-19”, which exemplifies expectations of good doctors [2, 3]. In effect, in addition to providing effective disease prevention, diagnosis, therapy, and rehabilitation, good doctors provide patients with physiological support and sufficient human care. However, standards for good doctor abilities vary according to the populations surveyed [4]. For instance, good communication and care-taking skills are deemed good-doctor abilities for patients; outstanding medical skills, trustworthiness, and integrity for peer doctors; being highly collaborative for nurses; generosity to share valuable medical knowledge and expertise for students; and the ability to meet key performance indicators for hospital executives.

“What is a good doctor? What abilities, attributes, and qualities should a good doctor possess? What effect will good-doctor abilities exercise?” We have seen many research efforts made on these topics; however, the questions are not easily answered [5]. Current literature focuses on the typical characteristics and behaviours of good doctors. A study in the UK outlined essential requirements for a good doctor, including recognition of patient care, probity (being honest, trustworthy and acting with integrity), compassion, good communication and listening skills, curiosity, creativity, the ability to be a team player, and potential for leadership [6]; a study by investigators in Israel upheld that good doctor qualities and behaviours are related to features of a humane relationship to patients, medical knowledge and skills, devotion and help to patients, good relations with staff, management and administration [7]; a study from South Korea proposed that good doctor attributes should include four themes—professional, academic-executive, personal value, and relationships [8]; a similar study from Mozambique pointed out that the characteristics of good doctors include being dedicated, good diagnostic and therapeutic skills, positive personal characteristics (including being calm, sympathetic, caring and supporting), respectful and nondiscriminating, skilled in communication (referring to listening and explaining) and others [9]. One Turkish paper listed good doctor attributes such as interpersonal relations and communication, sustaining professional integrity, scientific knowledge, and good medical practice [10]. The literature review above indicates that research on the characteristics of good doctors and good doctor behaviours can, to some extent, reflect good doctor abilities and connotations; however, more in-depth studies on the structure and effects of good doctor abilities and efforts to formulate complete theory remain lacking, and even more so are studies on what is happening in China.

China is home to 1.4 billion people, including 4.086 million physicians (2.9 physicians per 1,000 people) from specialties such as internal medicine, surgery, gynaecology, and paediatrics, all of whom support the largest medical system in the world [11]. A total of 7.74 billion visits to all medical institutions in China was reported in 2020 alone. Since June 2017, China’s central government has started to commend outstanding doctors and nurses who either have performed prominent undertakings when practising medicine, who have been working at the hospital frontline dedicatedly, who continue to improve public health in China, or who demonstrate well-respected moral competence [12] through an “Online Commendation Campaign of Chinese Good Doctors and Good Nurses”. The campaign selects 10 good doctors and nurses from nationwide each month and makes public their deeds on www.wenming.cn, the most credible online platform that commends individuals and organizations with exceptionally good deeds, remarkable contributions, or moral characters from all walks of life, drawing spectacular attention from the Chinese medical realm. Nomination process: The health department in each provincial region is responsible for nominating candidates, with elaborations on their deeds at the “Chinese Good Doctors, Chinese Good Nurses” platform for public review, before the final 10 good doctors and nurses are selected.

Thematic content analysis was applied in this study to analyse the good deeds and relevant information of good doctors in China on this website and accordingly successfully constructed the “structure and effects of the good-doctor abilities in China” model, which can answer two questions—“what abilities should a good doctor in China possess” and “what effects will good-doctor abilities exert”—in an attempt to diversify relevant theories, facilitate discussions on how to achieve better working performance and offer experiences and theories from China to enhance medical education worldwide.

## Methods

### Study design

Thematic content analysis is a method for analysing qualitative data; it is commonly used for empirical material and texts such as interviews or textual notes and was originally created by Braun and Clarke [13]. This flexible method, a six-stage architecture consisting of familiarization, initial coding, generating themes, reviewing themes, defining and naming themes, and producing the report, can be adapted to different research types [14]. Since the current literature available in China on this topic still falls short, this study established itself by thematic content analysis, aiming to explore a new phenomenological framework in the structure of good doctor abilities and the corresponding effects.

### Data collection

The “Good Doctors and Nurses in China” Commendation Campaign has been quite influential domestically, as it allows many unknown doctors with remarkable contributions and accomplishments to be seen, honoured, and appreciated. In each session, the winning doctors and nurses will be published on www.wenming.cn. This study collected 50 Chinese good doctors selected from January to July 2021 in chronological order from the website as research samples, excluding nurses during the same period. Accordingly, their deeds were analysed, and empirical descriptions regarding ability structure and effects were obtained. To ensure data fidelity and representativeness, broad sampling was implemented: 50 doctor participants were included from both private and public hospitals in 9 provincial regions in distinct locations (Beijing, Liaoning, Shandong, Fujian, Guangdong, Guangxi, Hainan, Shanxi, and Chongqing), covering rural and urban areas.

### Data analysis

The personal profiles and outstanding deeds of the doctor subjects were entered into Excel sheets, and the following criteria were used: gender, age, education, work experience, specialty, and good deeds. When there was insufficient information, the investigators perused the official websites of the hospitals where the doctors worked to complete the information collection. It took one month to complete the data collection and input process, and 42,000 Chinese character documents were generated. Before analysis, investigators filtered and crosschecked the data to ensure that they could accurately represent the characteristics of the doctors under study.

**Fig. 1:**
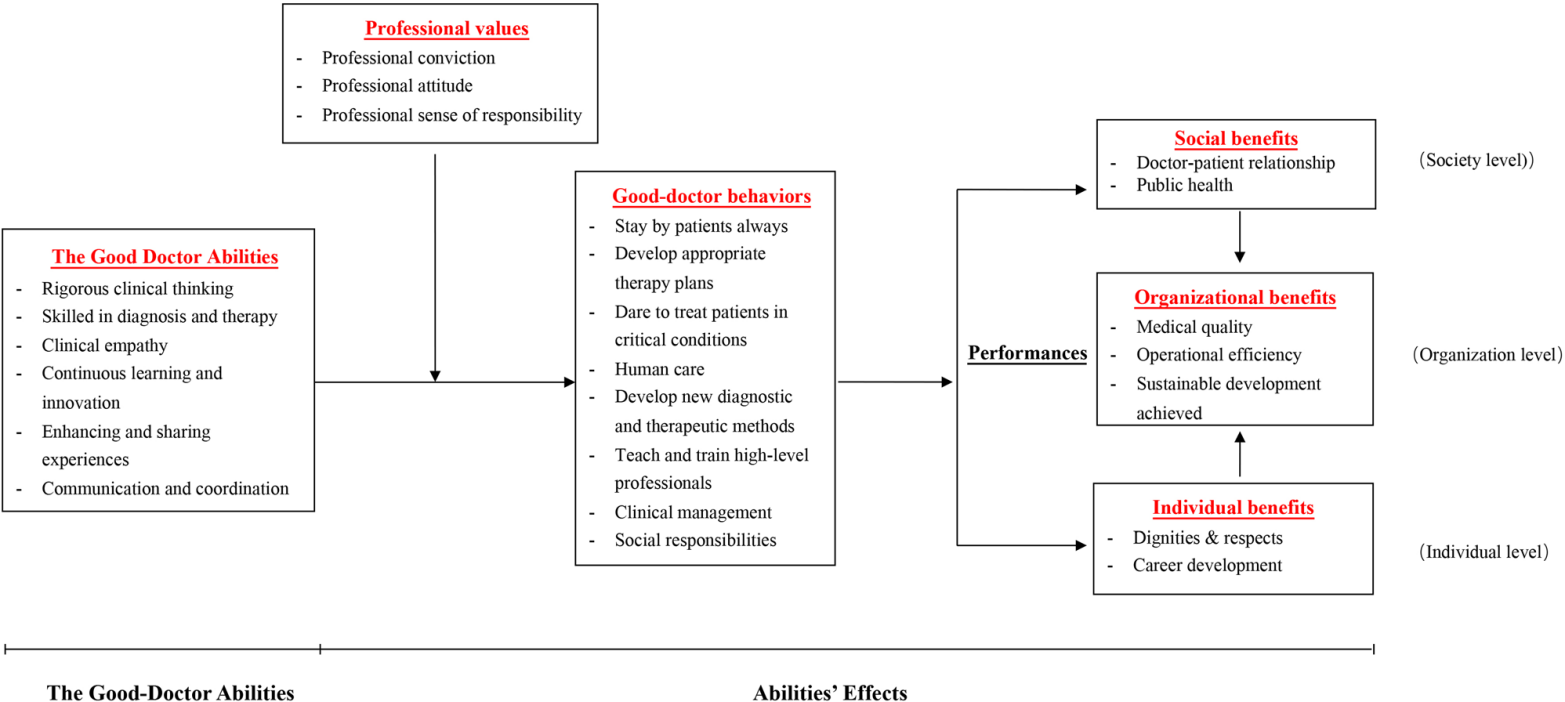
Model of the structure and effects of good doctor abilities in China

After the above steps, we proceeded to the data analysis stage. In the first step, the personal stories of 50 outstanding doctors were repeatedly read, with notes taken during the reading process to ensure a high level of familiarity with the data; the second step involved coding the content relevant to the research question in the text materials. The coding team consisted of three members familiar with the field. Through group discussions, the original data were collaboratively coded. NVivo11 software was used for organizing, coding, and analysing the text data. A total of 84 codes were identified, including categories such as “diagnosis and differential diagnosis” and “analysis and reasoning”. In the third step, the process of finding themes involved categorizing the codes from the previous step into higher-level themes and analysing the logical relationships between different codes and themes, resulting in 24 initial themes. The fourth step included a review of the themes, where all authors of the paper extensively compared and analysed the 24 initial themes, made modifications and eliminated certain themes that were similar or contradictory, ultimately resulting in 6 themes. The fifth step focused on finalizing and naming the 6 themes, clearly defining the content of each theme, as shown in Table 1. The final step involved writing the final report and creating a new theoretical framework to address the scientific questions posed in this study, as shown in Fig. 1.

**Table 1:** The process of thematic content analysis

Themes	Subthemes	Code examples
Good doctor abilities	Rigorous clinical thinking; Skilled in Diagnosis and therapy; Clinical empathy; Continuous learning and innovation; Enhancing and sharing experience; Communication and coordination	Clear decision-making mindset; diagnosis and differential diagnosis; Data analysis and reasoning; Rich clinical experience; Comprehensive medical knowledge, etc.
Professional values	Professional conviction; Professional attitude; Professional sense of responsibility	Firm medical ideal; Job satisfaction; Love medical career; Doctor’s mission, etc.
Good doctor behaviour	Stay by patients always; Develop appropriate therapy plans; Dare to treat patients in critical conditions; Human care; Develop new diagnostic and therapeutic methods; Teach and train high-level professionals; Clinical management; Social responsibilities	Patient-centred; help rural patients; sticking to clinical frontline; Precision treatment measures; Clinical suitability; Treatment of emerging infectious diseases, etc.
Social benefits	Doctor‒patient relationship; Public health	Patient trust; Dissemination of health concepts; Ensuring the health of the poor, etc.
Organizational benefits	Medical quality; Operational efficiency; Sustainable development	Improving clinical efficacy; Establishing a quality control system; Improving work efficiency, etc.
Individual benefits	Dignities and respects; Career development	International recognition; Academic influence, etc.

### Study results

#### Demographic profile

The ages of all 50 doctor subjects under investigation ranged from 37 to 93 years, among whom 42 were male (84%), 8 were female (16%), 28 were between 51 and 60 years (56%), 15 were between 41 and 50 years (30%), 18 were from the Internal Medicine Department (36%), 11 were from the Surgery Department (22%), and 6 were from the General Practice Department (12%). In terms of service years, 23 doctors had between 31 and 40 years of service (46%), and 14 had between 21 and 30 years of service (28%). Regarding education levels, 31 held a master’s degree and above (62.00%), and 14 held a bachelor’s degree (28.00%). See Table 2 for more details.

**Table 2:** Demographic characteristics of the doctors under investigation

Characteristics	Variable	Frequency (N)	Percentage (100%)
Gender	Male	42	84.00
	Female	8	16.00
Age	31–40	2	4.00
	41–50	15	30.00
	51–60	28	56.00
	> 60	5	10.00
Specialty	Internal Medicine	18	36.00
	Surgery	11	22.00
	General practice	6	12.00
	Intensive care medicine	5	10.00
	Preventive Medicine	3	6.00
	Traditional Chinese Medicine	3	6.00
	Paediatrics	2	4.00
	Psychiatry	1	2.00
	Clinical radiology	1	2.00
Service year	11–20	10	20.00
	21–30	14	28.00
	31–40	23	46.00
	> 40	3	6.00
Education level	Below Bachelor’s Degree	5	10.00
(in Medicine)	Bachelor’s Degree	14	28.00
	Master’s Degree	10	20.00
	Doctor’s Degree	21	42.00

### Study findings

This study attempted to reflect the features of good doctors in China and innovatively proposed the “structure and effects of the good-doctor abilities in China” model. The independent variables are good doctor abilities, the dependent variables are effects/performance on three levels (social benefits, organizational benefits, and individual benefits), the mediator variables are good doctor behaviours, and the moderator variables are professional values. Figure 1 presents more details.

Within this model, 24 subthemes were found, namely, “rigorous clinical thinking”, “skilled in diagnosis and therapy”, “clinical empathy”, “continuous learning and innovation”, “enhancing and sharing experience”, “communication and coordination”, “professional conviction”, “professional attitude”, “professional sense of responsibility”, “stay by patients always”, “develop appropriate therapy plans”, “dare to treat patients in critical conditions”, “human care”, “develop new diagnostic and therapeutic methods”, “teach and train high-level professionals”, “clinical management”, “social responsibilities”, “doctor‒patient relationship”, “public health”, “medical quality”, “operational efficiency”, “sustainable development”, “dignities and respects”, and “career development”. All of these categories are embodied in 6 core themes according to the attributes of each category—“good doctor abilities”, “professional values”, “good doctor behaviours”, “social benefits”, “organizational benefits”, and “individual benefits”. The underlying logic of this model is that “driven by professional values, good-doctor abilities render more good-doctor behaviours, and thus create benefits on social, organizational and individual levels through generating social benefits, improving organizational performance of the hospital and bettering individual performance of the doctor ”, thus clearly responding to two essential questions- “what the good-doctor abilities are” and “what their effects are”.

### Structure of the good doctor abilities in China

In this study, we examined the structural dimensions of good doctor abilities from the perspective of competence and quality as follows:

#### Rigorous clinical thinking

This ability plays an integral role in good doctors and is the foundation for them to comprehensively perceive, analyse and resolve medical problems [15, 16]. It is mainly demonstrated by putting patients first in all medical routines, such as recording disease history and conducting all necessary physical examinations/laboratory examinations; following a clear-cut and thorough decision-making process in diagnosis through analysing, reasoning, comparing, verifying, and identifying the symptoms, based on all shreds of evidence and information gathered at hand; working together with patients’ family members; and fully considering the living environment of the patients to determine the most appropriate diagnosis and therapy plan.

He in the first place, obtained an understanding of the specific condition of the patient, checked the patient’s various physical signs such as the vessels of tongue, and conducted four-step basic examination by inspecting the appearing features, listening to the heartbeats, voice and breathing noise, asking questions, and inspecting the pulses for each patient. Afterwards, he summarized his observations with a primary diagnosis of suspected symptoms in a timely manner, analysed the syndrome characteristics of COVID-19 patients, and was guided to treat mild, moderate, and asymptomatic infected patients accordingly from a traditional Chinese medicine perspective.” Sample 28.

#### Skilled in diagnosis and therapy

Additional crucial components for good doctors include diagnostic, therapeutic, and technical skills; competence and accuracy; knowledge; and expertise required to mitigate afflictions and treat and cure diseases [17, 18], which mainly consist of a good command of edge-cutting knowledge in medicine and complicated diseases; familiarity with ethics, customs, and manners of different cultures and backgrounds; rich clinical experiences; medical skills for difficult-to-treat diseases and crucial conditions; and the ability to make accurate diagnostic and therapeutic plans for challenging cases.

A young girl suffering from severe thyroid cancer sought him out and begged for surgery to be performed. However, due to the young age of the child, there were significant risks associated with surgery. If the surgery failed, it could endanger the patient’s life. Nevertheless, he ultimately withstood immense pressure and successfully underwent surgery on the young girl. The patient recovered well after the surgery, demonstrating the doctor’s ability to save lives.” Sample 27.

#### Continuous learning and innovation

Medicine is a fast-evolving discipline that requires life-long learning and dedication to push boundaries amid enormous clinical challenges; otherwise, it will stagnate. As a good doctor, he or she should be committed to clinical work, always stay by the patients, and keep in step with the latest clinical guidance and latest research on rare or hard-to-treat diseases through lifelong learning, reading, and constantly attending professional development programs [19]; at the same time, he or she should take a rigorous, meticulous, and ever-upwards attitude to approach clinical problems, undertake in-depth research and innovation, reveal the features of difficult diseases, and transform research findings into clinical skills [20, 21].

He was the first in China to have introduced a series of cutting-edge international technologies such as magnetic resonance simulation positioning, transforming cancer radiation therapy in China from a conventional method to more precise ones. The research outcomes have been adopted in cancer diagnosis and treatment guidelines both domestically and internationally and have been acclaimed by top academic journals as “revolutionary technology and groundbreaking innovation.” Sample 17.

#### Clinical empathy

Social psychologists defined “empathy” as the capacity to understand or feel what another person is thinking or experiencing from within their frame of reference [22]. Clinical empathy is exhibited by two aspects: on the one hand, good doctors possess relatively strong empathy and benevolence [23, 24], reverence for life, and care for patients, all of which constitute the premises of being compassionate-empathetic; on the other hand, in medical practices, they will be able to understand patients’ suffering, take a mild and amicable manner to interact, maintain sufficient communication, think from the perspective of the patient, be meticulous and tolerant, be willing to listen, give human care, and others to achieve the best therapeutic results and create a harmonious doctor‒patient relationship.

She often says, The archenemy of a doctor is Indifference, and the most effective prescription is kindness and caring. As long as your attitude improves even by the least bit, it may change a patient’s life. Even a small gesture of kindness from a doctor can sow a ray of sunshine in the patient’s heart.” Sample 41.

#### Enhancing and sharing experience

A good doctor should be able to address complex clinical issues with operable medical frameworks summarized from clinical findings and worthwhile cases, locate the potential value of these findings worthy of further study, and be open to sharing with medical students and colleagues [25, 26]. In this process, two good doctor qualities are needed: (1) to continuously reflect and analyse the clinical cases and lessons learned, summarize the common rules out of complex clinical issues, and carry the findings to higher theoretical and practical levels, thus framing a clinical experience and knowledge architecture with personal characteristics; (2) to have a strong sense of responsibility in applying all personal experiences and knowledge into medical teaching, generously share valuable knowledge with future generations with flexible and diversified teaching methods, and pave the way for medical professionals’ growth and medical advances.

“*He offered teaching sessions, standardized medical record writing, taught medical techniques to clinicians from community-level hospitals in western China, and generously passed advanced medical knowledge and concepts on to medical workers in western China through personal examples and precepts. Improved medical services at local hospitals. ” Sample 21*.

#### Communication and coordination

A good doctor should have good communication, coordination, and presentation skills and be able to communicate and collaborate effectively with patients, family members, caregivers, and other medical professionals. It mainly refers to the ability to implement team spirit in the handling of doctor‒patient relationships and staff relations through communicating and coordinating with all stakeholders concerned and to lead the team to effectively and efficiently meet key performance indicators through good planning, execution, coordination and supervision [27, 28].

During the early stages of the COVID-19 outbreak, carrying out standardized treatment was a challenge for the medical team. He took the initiative to assume the responsibility of team management and operation, had effective communication with the hospital medical department in the short term, organized the writing and formulation of work processes, and implemented a grouped business operation model to solve problems that arose for everyone.” Sample 15.

### The effects of good doctor abilities in China

Appelbaum proposed the AMO Model for high-performance work systems, asserting that improvements in personal knowledge, skills, and capabilities could directly improve individual and organizational performance [29]. This study applied the “Ability-Motivation-Opportunity” (AMO) theory to depict the multidimensional effects of good doctor abilities on aspects of society, organizations, and individuals:

#### Social benefits

Some researchers mentioned that good doctors ought to “promote and protect the health of both patients and the public” and require doctors to provide health care to a broader population [30]. Good doctor abilities will create social benefits by encouraging doctors to engage in more good doctor behaviours, such as promoting and improving doctor‒patient relationships [31, 32], winning trust from patients and their family members, and building a positive image through media reports, all of which will be conducive to creating harmonious doctor‒patient relationships and promoting broader respect for doctors across society. Good doctors are obligated to disseminate medical knowledge to the broader population, raise public awareness and knowledge on health care, and take part in poverty reduction schemes by promoting the health of underprivileged people and empowering the medical capability of impoverished areas; they also shoulder the responsibilities of making health care-related suggestions to the government, facilitating and proactively engaging in health care-related policy-making.

#### Organizational benefits

Good-doctor behaviours play a crucial role in hospital development, mainly through three aspects—raising a hospital’s medical quality, boosting operational efficiency, and promoting sustainable development—in terms of medical quality, good doctors could help standardize clinical practices by writing or cowriting official guidelines and continuously upgrading medical quality and clinical therapeutic efficacy with the implementation of a better quality management and control system [33]. In terms of boosting operation efficiency, good doctors will ensure that current medical resources meet the needs of the maximum number of patients by elevating medical efficiency and increasing the number of patients served or other effective organizational management approaches [34]. In terms of sustainable development, good doctors will intensify input to the development programs of medical professionals and wisely designed professionals’ echelons, achieve academic fruitions by delivering innovative breakthroughs, and expedite the quality development of and proliferation of the academic standing of the specialty and the hospital.

#### Individual benefits

Good doctor work performance and behaviours have significant influences on career development, including respect and career advancement. As for the former, good doctors acquire respect after winning the trust of patients and their family members [35] for providing satisfying medical service, obtaining recognition from peer doctors and hospitals for excellent medical skills and/or major academic breakthroughs, and being granted government recommendations owing to their meritorious role and eminent contribution to social causes. For the latter, good doctors will build a stronger academic profile of their hospital due to the accomplishments mentioned above or by assuming high-ranking executive roles in hospital or trade associations with outstanding management charms, versatility, and team spirit.

## Discussion

### Profoundly mapped out good doctor abilities and behaviours and depicted “what a good doctor in China should be like”

To date, no consensus on a good doctor’s definition has been reached across academia. This study, with the help of the Model of Abilities Structure and Effects, has set a pioneering step for studies of its kind.

First, six ability categories are proposed. The rapid growth of disease spectrum and medicine R&D has led society to place greater demands on doctors’ professional skills and knowledge. In this sense, the need to build a multidimensional abilities structure that encapsulates medical therapy, education, research, and management became highly necessary. This article systematically illustrates the abilities structure and proposes six essential ability categories suitable for current China, which are different from those proposed by the Medical Schools Council of the UK (MSC), the Accreditation Council for Graduate Medical Education (AGGME) of the US, and the Canadian Medical Education Directives for Specialists (CanMEDs) of Canada, since these three institutions are oriented towards different priorities for cultivating resident physicians. Therefore, their attribute structures were mainly used in coaching or training plans for physicians. In China, the standards that define good doctors are rooted in how the public perceives whether or not a doctor is good, and these standards are correspondingly implemented in the education and selection of medical professionals.

Second, the results of this study profoundly reflected good doctor behaviours in China. Previous studies focused on good doctor attributes but rarely focused on good doctor behaviours. This article attempted to do both, maintaining that conducting good clinical practices is the central mission for good doctors, and they should be committed to handling disease with dedication, such as staying by patients always, providing accurate diagnosis and effective therapy, providing human care and daring to take on hard-to-treat diseases or critical conditions. However, good doctor behaviours should not be limited to clinical diagnosis and therapy but rather include a panoramic view of the whole health industry and scientific advances. For instance, they should invest more in research and innovations and improve clinical practices through continuous upgrading, updating, and innovating; emphasizing professionals’ education and upbringing, especially in exceptionally important specialties with great potential for scientific advances and innovative breakthroughs, to achieve sustainable development and renew professionals’ echelons. From a broader perspective, efforts must be made to improve the entire health care system [36] by improving health services; and promoting social progress and medical development through fulfilling social responsibilities, such as participating in “poverty reduction initiatives through health improvement”, conducting science outreach campaigns, and engaging in public emergency relief and rescue.

Third, this research presented the professional values of good doctors, which reflect the personal career goals and attitudes of doctors. These values refer to doctors’ orientation and behaviours at work, as well as the conviction and attitude towards the career they choose [37]. In the workplace, good doctors are required to bear a hefty sense of value. Only with such, can they put the patient first and service them wholeheartedly. In particular, professional conviction refers to an unwavering faith to practice medicine and commit to alleviating patients’ suffering; a professional attitude signifies a passion towards their work and dedication, perseverance, resilience, meticulousness, diligence, excelsior, and selflessness while they work; and professional responsibility refers to a positive mental state willing to perform all possible good deeds both within and outside their responsibility sphere to benefit themselves and others. For example, amid the COVID-19 outbreak, medical workers from all over China worked selflessly, sacrificed their health to save lives, and fought against the disease, which best exemplified the sense of responsibility found in good doctors and set the example of positive professional value to the wider world.

### Carrying forward the study of the effects of and pathways to be a good doctor

The AMO framework of “ability-motivation-opportunity to participate” was applied to identify the study logic, which is “the good-doctor abilities – improve good-doctor behaviours – exert positive impacts on good-doctor personal development and create social benefits – better the organizational wellbeing and performance of the hospital”, thereby fulfilling the untouched ground in good-doctor research. According to the findings of the article, good doctors need to deal with multiple stakeholders throughout the whole medical system, including patients, coworkers, hospitals, trade associations, governments, and other people and organizations, and the influence of good doctors is on many more aspects than just their career development. For example, many studies have validated the correlation between good doctor abilities and patient satisfaction [38], similar to the mechanism by which good doctor abilities impact society, organizations, and individuals. This study took steps to further systematically elaborate the effects on these three levels and discussed the pathways needed to achieve high performance by a good doctor. Specifically, they are as follows.

### Pathway one: good doctor abilities promote good doctor behaviours and then result in better social, organizational and individual performance

Having six good doctor abilities is a prerequisite for being a good doctor and the driving force for delivering more high-performance behaviours, especially in clinical scenarios. Good doctor behaviours can be summarized as clinical practices, innovations, teaching and training, medical management, and social responsibilities. In particular, they are as follows: (1) clinical practices are identified as behaviours of staying by patients always, daring to take on hard to treat diseases or critical conditions, developing appropriate therapy plans and offering enough care to patients; (2) innovation for developing new diagnostic and therapeutic approaches by working on arduous medical agendas; (3) teaching and training signify providing guidance, teaching postgraduate students, and training new doctors to ensure their sound growth for higher medical accomplishments; (4) medical management refers to voluntarily assuming the executive role of the team within the department and hospital by displaying leadership and sense to coordinate among multiple stakeholders, as well as formulating and implementing more efficient management strategies; and (5) social responsibilities indicate handling various public health emergencies or emerging infectious diseases, participating in social programs for poverty alleviation through health promotion programs initiated by all levels of governments, as well as offering advice and suggestions to the government on making health care-related policies.

Through good clinical practices and honouring social responsibilities, trust from patients, their family members, and even society as a whole will be acquired; thus, a harmonious doctor‒patient relationship, improved public health, and more social benefits will be created; through good medical management, innovations, and education, the service quality, medical security and operational efficiency of the hospital will be ensured; thus, the high quality and sustainable development of the hospital will be achieved. By embedding medical innovations into clinical practices, good doctors will eventually harvest academic fruitions, win respect across society, and achieve greater success in their careers.

### Pathway two: professional values will have a positive impact on good doctor abilities and behaviours

The professional values of good doctors strengthen their conviction to rescue the dying and heal the wounded, ensure that their behaviours are in line with their aspirations, and enhance their professional morality, passion, dedication, and devotion to medicine. Driven by such a strong value, good doctors will focus more intensively on all the endeavours mentioned above by sharpening their abilities to do so and perfect their skills and competence; moreover, professional values will increase their sense of gain, help them recognize their contribution to medical causes, and continuously propel them to engage in more high-performance behaviours [39] to maintain an unswerving commitment to making innovations and breakthroughs, love their work, and improve their health.

Traditional Chinese medicine culture and long-standing moral values also play important roles. For centuries, Chinese doctors have upheld the philosophy that “benevolence and an empathetic heart is the priority for being a doctor”. Sun Simiao, a renowned doctor from the Tang Dynasty (618 A.D.–907 A.D.) dubbed the “King of Traditional Chinese Medicine”, pointed out that “be they senior or junior, rich or poor, intimate or strange, pleasant looking or repulsive, smart or retarded, friendly or hostile, all patients must be treated with a kind heart.” [40] These values characterized by Chinese morality have been the underlying features in the value system of many Chinese doctors and will continue to play a part in shaping Chinese good doctors.

### Study limitations

This study utilized thematic content analysis to interpret and analyse the textual data of “good doctors”, which includes some unobtrusive data. However, due to the influence of researchers’ personal subjective views, bias may exist in the research results; moreover, limited by the quality of the text data, the research content may not cover all relevant texts, which may lead to incomprehensiveness of the results. To address these issues, future research could focus on the following aspects from a microtheorizing perspective. We recommend performing interviews to understand the genuine attitudes and opinions of good doctors; identify the objectivity limitations of text analysis and make necessary complements; conduct on-site research to collect first-hand data to improve the reliability and effectiveness of the research; conceptualize the variables involved in the model of this study through empirical research; develop measuring tools; and use large-scale surveys to validate the relationships among variables in the model [41].

## Conclusions

This study took root in China to propose the “Structure and Effects of the Good Doctor Abilities in China” model and produced detailed interpretations by thematic content analysis based on the good deeds of 50 good doctors. The abilities of the good doctor consist of six categories: rigorous clinical thinking, skilled in diagnosis and therapy, clinical empathy, continuous learning and innovation, enhancing and sharing experience, and communication and coordination. All of these factors exert positive impacts on the performance and development of hospitals since they place a beneficial force on the personal growth of doctors and benefits on society. This model consists of six core categories, including good doctor abilities, professional values, good doctor behaviours, social benefits, organizational benefits, and individual benefits. The independent variables are good doctor abilities, the dependent variables are performance on three levels (society, organization, and individual), the mediating variables are good doctor behaviours, and the moderating variables are professional values. This model responds to two questions: “What abilities should the good doctor in China possess?” and “What effects will the good doctor abilities exert?”. These findings can contribute to good doctor ability theories and similar research and are a theoretical innovation for global health care-related studies.

These study results also realize pragmatic significance for medical education worldwide and provide a performance review of doctors. On the one hand, this model lays the foundation for properly conducting medical education and training. In medical practice, pedagogical experts could design new review and evaluation systems or courses to promote professional abilities, professional values, and good doctor behaviours guided by this abilities structure; conduct review, evaluation, and training for medical students and resident doctors; assess their competence in the posts; and intensify well-targeted training in areas that are still lacking to increase the professionalism and knowledge of doctors as well as cultivate more good doctors. On the other hand, this model offers a reference for the performance review of medical workers. Medical institutions could follow this model and its criteria to establish a more science-based and applicable review and evaluation system oriented towards medical professionals and adopt a more human-centred and targeted education and management approach to enhance the comprehensive abilities of clinical doctors. Moreover, this model could also provide a theoretical reference for the recruitment of clinical doctors, the design of training programs for new doctors, and the development of review, evaluation, and rating systems to address the need to cultivate high-quality professionals and improve human resource management in hospitals.

### Electronic supplementary material

Below is the link to the electronic supplementary material.

Supplementary Material 1

## Data Availability

All the data used in this study are publicly available. The website is www.wenming.cn.

## Abbreviations

AMO: Ability Motivation and Opportunity Theory
WHO: World Health Organization
UK: United Kingdom
