# Supplementary material for: A thematic content analysis of the structure and effects of good doctor abilities in China
Source: BMC Health Serv Res. 2024 Jul 16;24:819. doi: 10.1186/s12913-024-11145-2 (PMC11253447; doi:10.1186/s12913-024-11145-2)
Supplement: Supplementary file 1 — Supplementary Material 1 [file 12913_2024_11145_MOESM1_ESM.pdf]

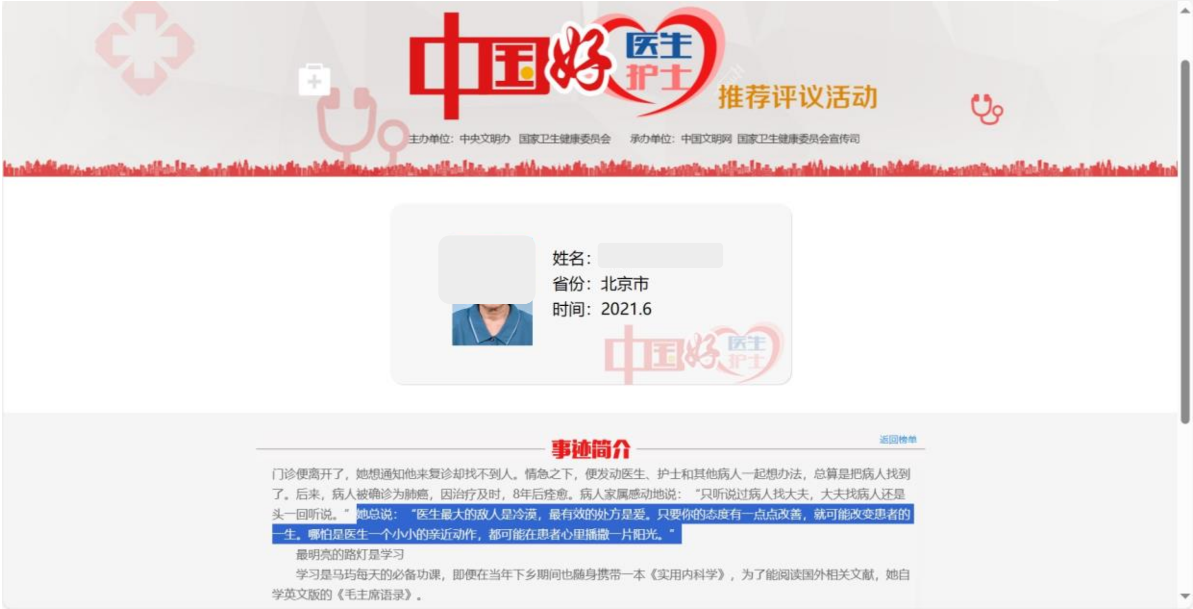

We used a case to illustrate the process of labeling.

事迹简介

最有效的处方是爱（Love is the most effective prescription）

每次听诊前，她会用手先把听诊器捂热；听完前胸听后背时，她会自己走到患者背后。每次给老年患者做完检查，她总要扶着对方下了诊查床再去开处方……半个多世纪过来，这些细微得有点琐碎的行医习惯在马医生看来，再普通不过了。“病人是弱势群体，做医生的不能高高在上。医生只有坚持平等待人的原则，才能给病人以尊严。”这就是马医生的行医风格。

曾经一位病人右下肺发现病灶，来到医院求治。马医生觉得不太像结核，需要观察一段时间。然而，病人看过门诊便离开了，她想通知他来复诊却找不到人。情急之下，便发动医生、护士和其他病人一起想办法，总算是把病人找到了。后来，病人被确诊为肺癌，因治疗及时，8年后痊愈。病人

批注 [A1]: latent central profile

Original Text translation: Before each auscultation, she warms the stethoscope with her hands; after listening to the front and back of the chest, she walks to the back of the patient herself. After examining elderly patients, she always helps them off the examination bed before writing the prescription.

This passage describes the process of a doctor examining a patient, but every action and detail she takes demonstrates love for the patient. This is a latent, hidden expression, yet it carries significant meaning at the core.

批注 [A2]: peripheral profile

Original Text translation: Once a patient found lesions in the right lower lung, came to the hospital for treatment. Dr. Ma doesn't think it looks like tuberculosis and needs to be observed for a while. However, the patient left the clinic and she wanted to inform him to come back but no one could be found.

This passage describes a doctor seeing a patient in the outpatient clinic who doesn't seem to have tuberculosis, but she wants to schedule a follow-up appointment with the patient. It shows her concern for the patient, although she doesn't directly express love for the patient, it is related.

家属感动地说：“只听说过病人找大夫，大夫找病人还是头一回听说。”

她总说：“医生最大的敌人是冷漠，最有效的处方是爱。只要你的态度有一点点改善，就可能改变患者的一生。哪怕是医生一个小小的亲近动作，都可能在患者心里播撒一片阳光。”

批注 [A3]: manifest central profile

Original Text translation She often says, The archenemy of a doctor is Indifference, and the most effective prescription is kindness and caring. As long as your attitude improves even by the least bit, it may change a patient's life. Even a small gesture of kindness from a doctor can sow a ray of sunshine in the patient's heart.

This passage fully expresses the love of the doctor for the patients, directly related to the core content or theme.
